# A multi-Task Learning based applicable AI model simultaneously predicts stage, histology, grade and LNM for cervical cancer before surgery

**DOI:** 10.1186/s12905-024-03270-1

**Published:** 2024-07-26

**Authors:** Zhixiang Wang, Huiqiao Gao, Xinghao Wang, Marcin Grzegorzek, Jinfeng Li, Hengzi Sun, Yidi Ma, Xuefang Zhang, Zhen Zhang, Andre Dekker, Alberto Traverso, Zhenyu Zhang, Linxue Qian, Meizhu Xiao, Ying Feng

**Affiliations:** 1 Department of Ultrasound, Beijing Friendship Hospital, Capital Medical University, Beijing, China; 2 Department of Obstetrics and Gynecology, Beijing Chao-Yang Hospital, Capital Medical University, Beijing, China; 3 Department of Radiation Oncology (Maastro), GROW-School for Oncology, Maastricht University Medical Centre+, Maastricht, The Netherlands; 4 Institute for Medical Informatics, University of Luebeck, 23562 Luebeck, Germany; 5 German Research Center for Artificial Intelligence, (DFKI), Lübeck, Germany

**Keywords:** Cervical cancer, Diagnosis, Prediction, ANN, AI, Preoperative, Prospective, Multi-task learning

## Abstract

**Purpose:**

To build an Mult-Task Learning (MTL) based Artificial Intelligence(AI) model that can simultaneously predict clinical stage, histology, grade and LNM for cervical cancer before surgery.

**Methods:**

This retrospective and prospective cohort study was conducted from January 2001 to March 2014 for the training set and from January 2018 to November 2021 for the validation set at Beijing Chaoyang Hospital, Capital Medical University. Preoperative clinical information of cervical cancer patients was used. An Artificial Neural Network (ANN) algorithm was used to build the MTL-based AI model. Accuracy and weighted F1 scores were calculated as evaluation indicators. The performance of the MTL model was compared with Single-Task Learning (STL) models. Additionally, a Turing test was performed by 20 gynecologists and compared with this AI model.

**Results:**

A total of 223 cervical cancer cases were retrospectively enrolled into the training set, and 58 cases were prospectively collected as independent validation set. The accuracy of this cervical cancer AI model constructed with ANN algorithm in predicting stage, histology, grade and LNM were 75%, 95%, 86% and 76%, respectively. And the corresponding weighted F1 score were 70%, 94%, 86%, and 76%, respectively. The average time consumption of AI simultaneously predicting stage, histology, grade and LNM for cervical cancer was 0.01s (95%CI: 0.01–0.01) per 20 patients. The mean time consumption doctor and doctor with AI were 581.1s (95%CI: 300.0-900.0) per 20 patients and 534.8s (95%CI: 255.0-720.0) per 20 patients, respectively. Except for LNM, both the accuracy and F-score of the AI model were significantly better than STL AI, doctors and AI-assisted doctors in predicting stage, grade and histology. (*P* < 0.05) The time consumption of AI was significantly less than that of doctors’ prediction and AI-assisted doctors’ results. (*P* < 0.05

**Conclusion:**

A multi-task learning AI model can simultaneously predict stage, histology, grade, and LNM for cervical cancer preoperatively with minimal time consumption. To improve the conditions and use of the beneficiaries, the model should be integrated into routine clinical workflows, offering a decision-support tool for gynecologists. Future studies should focus on refining the model for broader clinical applications, increasing the diversity of the training datasets, and enhancing its adaptability to various clinical settings. Additionally, continuous feedback from clinical practice should be incorporated to ensure the model’s accuracy and reliability, ultimately improving personalized patient care and treatment outcomes.

**Supplementary Information:**

The online version contains supplementary material available at 10.1186/s12905-024-03270-1.

## Introduction

Cervical cancer is still globally the 4th most common cancers in women. There were annually around 604 000 new cases and 342 000 deaths of cervical cancer worldwide [1]. Approximately 85% of new cases and 90% deaths occur in low-income and middle-income countries. Cancer survival for the most common cancers have improved since the mid-1970s except cervical cancer and uterine corpus cancer [2] Although cervical cancer is one of the most preventable cancers, it remains the second leading cause of cancer death among females aged 20 to 39 [3].

The cervix was the first organ of cancer clinical staging system designated by FIGO in 1958. Clinical evaluation is the first step in the assignment of cervical cancer stages. [4] The revised 2018 FIGO staging allow the use of available imaging and pathological resources to allocate the stage, which is close to the pathologic (TNM) staging. [5] But the prognostic factors, such as involvement of vascular/lymphatic spaces and isolated tumor cells should not change the staging. The lower staging is assigned when it is in doubt [4].

Actually, 8–42% of Stage IB–IVA cervical cancer patients showed para-aortic lymph node metastases [6]. Positive para-aortic lymph nodes have been reported in 35% of clinically evaluated stage IIB and 20% of stage III cervical cancer [7]. All these cases with lymph node metastasis (LNM) should be allocated to higher staging due to the worse prognosis. [8]. However, the staging and additional prognostic factors of cervical cancer guide the appropriate choice of the treatment [9–11]. his study aims to build an AI model on the basis of the postoperative TNM stage, which can precisely predict clinical stage, histology, grade and LNM at the same time, thereby enabling the selection of individualized treatment. Given the substantial impact of LNM on prognosis and treatment decisions, a model that can reliably predict these factors is crucial for improving patient outcomes. The complexity of integrating multiple predictive factors necessitates an advanced approach like multi-task learning (MTL) to capture the interdependencies and shared information among these tasks. Is it possible to use AI to predict stage, histology, grade and LNM for cervical cancer simultaneously before surgery?

Computer Aided Diagnosis (CAD) system has shown great performance with the rapid development of computer technology. Clinical decision support system (CDSS) is medical artificial intelligence (AI) and a system that help doctors with better clinical decisions, which can be used to prepare, review and filter diagnoses, and predict future events. [12–15] We previously used an AI model to predict histology, stage, and grade for endometrial carcinoma based on the preoperative examinations [16].

However, most of current machine learning task are designed on single task learning. When facing complex questions, it split the complex question into multi simple and independent subset questions and merge the results from the multiple single models to get the results of complex problem. However, lots of real-world questions cannot be divided into independent subset questions, that will overlook the shared information and relation between each subset questions. In this way, different the traditional signal task learning (STL) methods, multi-task learning (MTL) combines multiple related tasks together and learning multiple tasks at the same time.

Associative MTL can generalize better than STL by sharing the features between multiple tasks [17]. Artificial Neural Network (ANN) refers to the complex network structure formed by the inter-connectoin of a large number of units(neurons), which inspiration from the brain neural network [18]. It is a complex structure with a large number of simple elements connected to each other, which has a high degree of nonlinearity and can perform complex logic operations and nonlinear relationships.

In this article, we proposed a MTL based ANN network and designed a novel separate task loss function which make the model can be appropriate training for different tasks at the same time. Besides that, we design a Turing test to evaluate whether the AI has beneficial on the diagnosis of stage, histology, grade and LNM for cervical cancer.

The practical purpose of this study is to develop an AI model that can assist gynecologists in making accurate and rapid diagnostic decisions for cervical cancer, thereby improving patient care and outcomes. The integration of this AI model into clinical workflows can enhance the precision of diagnoses, reduce time consumption, and ultimately lead to more personalized and effective treatment strategies. The simultaneous prediction of stage, histology, grade and LNM before surgery has important clinical significance for the prognosis and treatment selection of cervical cancer, which helps to improve the patient’s tailored treatment and develop the most beneficial treatment plan for patients.

## Methods

### Study Subject

This study used a retrospective database of preoperative examinations in cervical cancer patients who were first treated in the Department of Obstetrics and Gynecology at Beijing Chaoyang Hospital, Capital Medical University between January 2001 to March 2014 [19] Moreover, we also prospectively collected a database of cervical cancer patients who received primary surgery in our hospital between January 2018 to November 2021 as independent validation set. Inclusion criteria: (1) undergoing surgical treatment at Beijing Chaoyang Hospital; (2) postoperative histopathology-confirmed cervical carcinoma; (3) with complete clinical-pathological data; (4) all treatments have been completed; (5) not pregnant at diagnosis. Exclusion criteria: (1) presence of primary malignant tumors of other organs, (2) metastatic cancer caused by malignant tumors of other organs, (3) not the first-time surgical treatment at Beijing Chaoyang Hospital, (4) pregnant at diagnosis, (5) incomplete clinical-pathological data. The study protocol was approved by the Institutional Review Board of our hospital and the patients’ information and statistics were anonymous and unidentified when analyzed. The obtained data included age, BMI, childbirth history, symptoms, physical examination, preoperative squamous cell carcinoma antigen (SCC), human papillomavirus (HPV) and Temperature Cycling Test (TCT) results, imaging results, menopause, postoperative histology, lymphatic metastasis, differentiation, stage based on the 2018 International Federation of Gynecology and Obstetrics (FIGO) staging system [20]. Ethics approval for this research was given by the Beijing Chaoyang Hospital, Capital Medical University.

### Data processing and machine learning algorithms

**Fig. 1:**
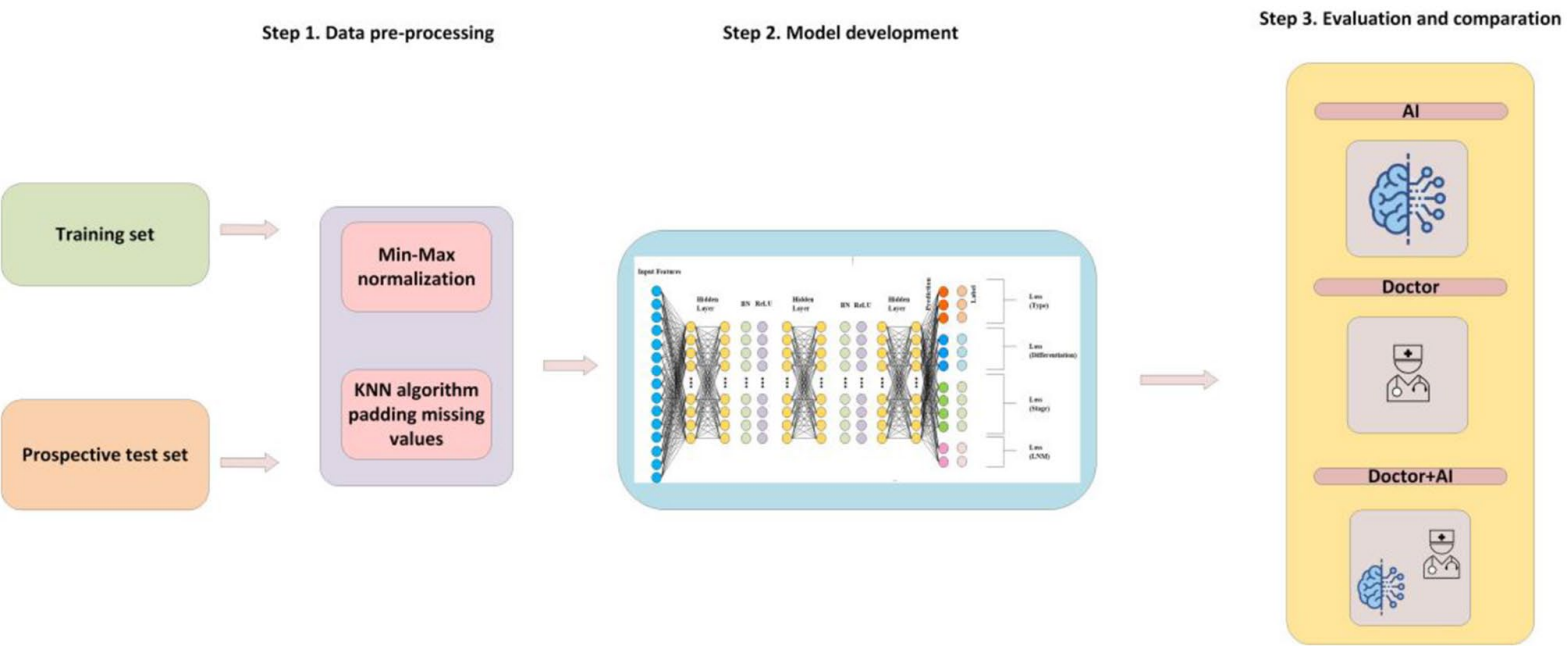
The workflow of this article

The whole workflow was shown in Fig. 1. For data preprocessing, first, total 14 features were selected for model training and prediction, according to guidelines. [4 -20 ] Second, the continuous features such as age and BMI were normalized by min-max normalization. Finally, the K-NearestNeighbor (KNN) algorithm (interpolation by the mean value of the nearest K data) was applied to fill the missing values.

**Fig. 2:**
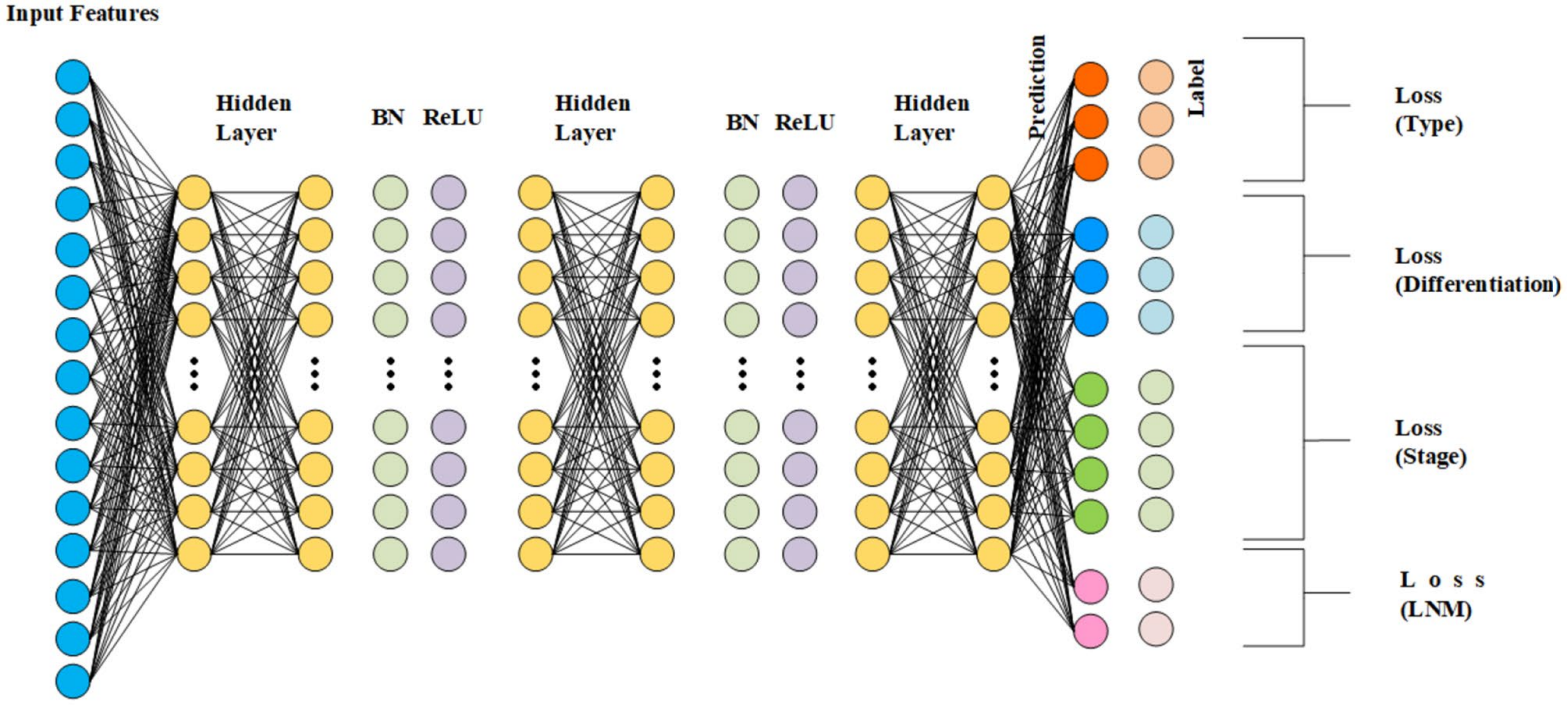
The structure of the Artificial Neural Network (ANN)

In this study, the ANN model composed of two fully connected layers (64 nodes) which have a Batch Normalization to protect gradient explosion and a Rectified Linear Unit (ReLU) activation function to increase the nonlinearity of the units and dropout layers with the rate of 0.5 to avoid over-fitting and one fully connected layer without activation function. The model structure was shown in Fig. 2.

Meanwhile, different targets/aspects of the disease are related in the process of disease diagnosis, so the diagnosis prediction of multiple targets/aspects can contain more comprehensive disease information than the diagnosis prediction of a single target. Therefore, we designed a loss function which can train the model to predict multiple targets/aspects. The function for loss were shown in Table 1, respectively. The Adaptive method (Adam) optimizer with a 0.001 learning rate was applied to train the model when changing the model weights.

**Table 1:** Loss function of the proposed method

$$\:\text{L}\text{o}\text{s}\text{s}\:=\:\frac{1}{\text{N}}\sum\limits_{\text{j}=1}^{\text{N}}(-\frac{1}{n}\sum\limits_{i}\sum\limits_{c=1}^{M}{y}_{ic}\text{log}\left({p}_{ic}\right))$$
N: number of task. n: sample size in each task. M: class number in each task. $\:{y}_{ic}$ : target label for each sample. $\:{p}_{ic}$ : prediction result for each sample.

The model was trained 100 epochs in the training set and test in the independent prospective test data based on the selected features to predict LNM, Grade, Stage and Type. On the other hand, to illustrate the advantages of MTL method, the comparison between the same models trained with proposed method and four STL method using cross entropy loss.

Data preprocessing was applied by pandas 1.4.2 and scikit-learn 0.24. The model was built by open source PyTorch 1.10 packages. All codes were written in Python 3.8 language.

In addition, to evaluate whether the AI is beneficial for clinical work. A Turing test was designed with gynecologists. A test set which independent with training set was separate into sub test sets (20 patients with/ without AI prediction results) and randomly send to doctors.

To evaluate the performance of the ANN model and Turing test performance, the accuracy and the weighted F1 score were calculated. F1 score was usually selected measure the accuracy of binary classification model. It takes into both precision and recall for the classification model. [21] Its maximum value is 1 and minimum value is 0.

The functions are shown below.

$$accuracy=\frac{TP+TN}{TP+TN+FP+FN}$$

$$\text{F}1\,\text{score}=\frac{2\,*\,\text{TP}}{2\,*\,\text{TP}+\text{FP}+\text{FN}}$$

$$\:weighted\:F1\:score\:=\:\sum\:_{i=1}^{C}\frac{{n}_{i}}{n}*{F1\:score}_{i}$$

TP: True Positive, TN: True Negative, FP: False Positive, FN: False Negative. C: the number of category. $\:{n}_{i}$ : number of samples with category is i. n: total samples.

### Statistical analysis

The comparison of accuracy, F-score and time consumption between AI model, doctor and doctor with AI were performed by using the ANOVA test in GraphPad Prism.

## Results

### Clinical information of cases

A total of 280 cervical cancer (CC) cases were retrospectively collected. Of these, 57 cases were excluded because of 70% or more of missing clinical data. Therefore, 223 cases were enrolled into the training set. The mean age was 41 (range 22–59) years old (Table 2. Clinicopathological data of patients with cervical cancer). The mean BMI was 24.1 ± 4.8 kg/m2. The gravida time and parity time is 3 (range 0–9) and 1 (0–5), respectively. Among these cases, 91% of the patients were squamous cervical carcinoma, and 7.2% of the cases were adenocarcinoma. The final FIGO stage of patients is verified based on the results of physical examination and postoperative pathology according to the 2018 FIGO Staging Classification of cervical cancer. 156 (70.0%) cases were FIGO stage I and 40 cases were stage II, 23 cases were stage III, and 4 cases were stage IV. About 53.8% of cases were grade (G) 1, 34 cases were G2, and 69 cases were G3.

**Table 2:** Clinicopathological data of patients with cervical cancer

Characteristics	Retrospective database	Prospective database
Number of patients	223	58
Age (y), mean (range)	41 (22–59)	48 (25–69)
BMI (kg/m^2^), mean ± SD	24.1 ± 4.8	24.0 ± 3.0
Gravida time, mean (range)	3 (0–9)	3 (0–7)
Parity time, mean (range)	1 (0–5)	1 (0–4)
FIGO stage ^a^		
I	156 (70.0%)	48 (82.8%)
II	40 (17.9%)	6 (10.3%)
III	23 (10.3%)	4 (6.9%)
IV	4 (1.8%)	0 (0%)
Histopathology type		
Squamous carcinoma	203 (91.0%)	44 (75.9%)
Adenocarcinoma	16 (7.2%)	9 (15.5%)
others	4 (1.8%)	5 (8.6%)
Differentiation		
G1	120 (53.8%)	21 (36.2%)
G2	34 (15.3%)	13 (22.4%)
G3	69 (30.9%)	24 (41.4%)

Data are expressed as mean ± SD or number (%) or number (range). BMI = Body Mass Index, calculated as weight in kilograms divided by height in meters squared; FIGO = International Federation of Gynecology and Obstetrics; G, grade; FIGO, the international federation of obstetrics and gynecology. a: FIGO stage, based on the results of physical examination and postoperative pathology according to the 2018 FIGO Staging Classification of cervical cancer

Furthermore, 60 CC cases were prospectively collected as independent validation set. Two cases were excluded because of incomplete clinical information. The mean age was 48 (range 25–69) years old (Table 1). The mean BMI was 24.0 ± 3.0 kg/m2. The gravida time and parity time is 3 (range 0–7) and 1 (0–4), respectively. Among these cases, 75.9% of the patients were squamous cervical carcinoma, and 9% of the cases were adenocarcinoma. 48 (82.8%) cases were FIGO stage I and 6 cases were stage II, and 4 cases were stage III. About 36.2% of cases were grade (G) 1, 13 cases were G2, and 24 cases were G3.

### The performance and turing test of model in prediction of stage, histology, grade and LNM for cervical cancer

The results of predictions are shown in Table 3. The performance of different models in prediction of stage, histology, grade and LNM for cervical cancer.

**Table 3:** The performance and turing test of models in prediction of stage, histology, grade and LNM for cervical cancer

Assessment	Accuracy				Weighted F1 Score			
Models	AIS	AI	DOC	DOC + AI	AIS	AI	DOC	DOC + AI
LNM	0.81(0.72–0.9)	0.76 (0.64–0.86)	0.80 (0.71–0.89)	0.80 (0.67–0.88)	0.84 (0.77–0.91)	0.76 (0.66–0.87)	0.78 (0.66–0.88)	0.77 (0.64–0.87)
Grade	0.63 (0.5–0.75)	0.86 (0.77–0.93)	0.63 (0.52–0.75)	0.68 (0.56–0.78)	0.62 (0.49–0.75)	0.86 (0.77–0.93)	0.6 (0.47–0.74)	0.66 (0.52–0.76)
Stage	0.69 (0.56–0.77)	0.75 (0.64–0.88)	0.62 (0.48–0.72)	0.67 (0.55–0.79)	0.68 (0.56–0.78)	0.7 (0.53–0.86)	0.53 (0.37–0.67)	0.6 (0.43–0.74)
Histology	0.81 (0.7–0.9)	0.95 (0.9-1.0)	0.89 (0.82–0.96)	0.83 (0.72–0.9)	0.78 (0.65–0.89)	0.94 (0.88-1)	0.89 (0.8–0.97)	0.86 (0.75–0.93)

*Notes* AIS: Single-Task learning based ANN model. AI, Multi-Task learning based ANN model; DOC, doctor’s Turing test; DOC + AI, doctor’s Turing test assisted with AI; LNM, lymph node metastasis

#### Stage

The accuracy for the STL AI, AI, doctor and doctor with AI were 0.69 (95%CI: 0.56–0.77), 0.75 (95%CI: 0.64–0.88), 0.62 (95%CI: 0.48–0.72) and 0.67 (95%CI: 0.55–0.79), respectively. The weighted F1 score for the STL AI, AI, doctor and doctor with AI were 0.68 (95%CI: 0.56–0.78), 0.7 (95%CI: 0.53–0.86), 0.53(95%CI: 0.37–0.67), and 0.6 (95%CI: 0.43–0.74), respectively. The AI has better performance than doctor and doctor with AI around 0.1. However, the STL AI has better performance than MTL AI model.

#### Histology

The accuracy for the STL AI, AI, doctor and doctor with AI were 0.81 (95%CI: 0.7–0.9), 0.95(95%CI: 0.9-1.0), 0.89(95%CI: 0.82–0.96), 0.83 (95%CI: 0.72–0.9), respectively. The weighted F1 score for the STL AI, AI, doctor and doctor with AI were 0.78 (95%CI: 0.65–0.89), 0.94 (95%CI: 0.88-1),0.89 (95%CI: 0.8–0.97), and 0.86(95%CI: 0.75–0.93), respectively. Both of AI, doctor and doctor with AI have achieve satisfactory performance over 0.8, better than STL AI.

#### Grade

The accuracy for the single-task AI, AI, doctor and doctor with AI were 0.63 (95%CI: 0.5–0.75), 0.86 (95%CI: 0.77–0.93), 0.63 (95%CI: 0.52–0.75), and 0.68 (95%CI: 0.56–0.78), respectively. The weighted F1 score for the single-task AI, AI, doctor and doctor with AI were 0.62 (95%CI: 0.49–0.75), 0.86(95%CI: 0.77–0.93),0.6 (95%CI: 0.47–0.74), and 0.66(95%CI: 0.52–0.76), respectively. The AI has better performance than doctor and doctor with AI and STL AI over 0.2.

### LNM

The accuracy for the STL AI, AI, doctor and doctor assisted with AI were 0.81 (95%CI: 0.72–0.9), 0.76(95%CI: 0.64–0.86), 0.80(95%CI: 0.71–0.89), and 0.80(95%CI: 0.67–0.88), respectively. The weighted F1 score for the STL AI, AI, doctor and doctor with AI were 0.84 (95%CI: 0.77–0.91), 0.76(95%CI: 0.66–0.87), 0.78(95%CI: 0.66–0.88), and 0.77(95%CI: 0.64–0.87), respectively. The doctor and doctor with AI and STL AI have a similar performance which was significantly better than AI.

The total average time consumption of STL AI and AI simultaneously predicting stage, histology, grade and LNM for cervical cancer was 0.01s (95%CI: 0.01–0.01) per 20 patients. The mean time consumption doctor and doctor with AI were 581.1s (95%CI: 300.0-900.0) per 20 patients and 534.8s (95%CI: 255.0-720.0) per 20 patients, respectively.

### Performance comparison between the AI model, doctors’ prediction and doctors’ prediction assisted with AI

**Fig. 3:**
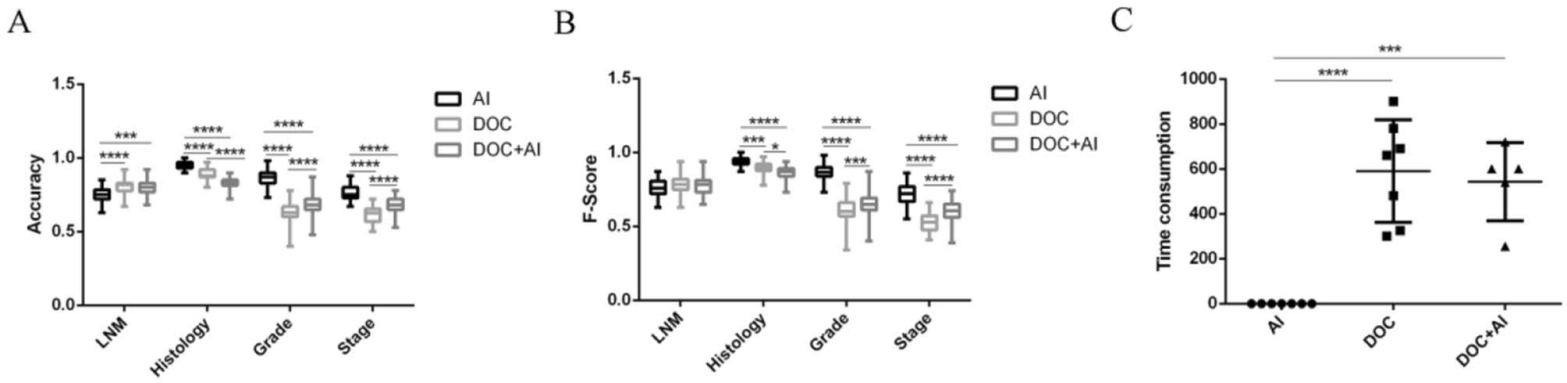
The comparison of Accuracy **(A)**, F-Score **(B)** and Time consumption **(C)** between

The comparison of the performance of AI model, doctor and doctor with assistance of AI is shown in Fig. 3. In this study, we organized an experiment involving 20 doctors: 8 resident doctors, 6 attending physicians, and 6 consultant physicians. These doctors were evenly divided into two groups, one with AI assistance and one without. Each doctor was required to evaluate 20 randomly selected patients, assessing them based on their clinical indicators. The group with AI assistance had access to AI-generated probabilities to aid their evaluations. Except for LNM, both the accuracy and F-score of the AI model were significantly better than doctors and AI-assisted doctors in predicting stage, grade and histology (*P* < 0.05). (Figure 3A and B) And the accuracy and F-score of doctors with AI assistance were significantly higher than that of doctor. In addition, the time consumption of AI is significantly less than that of doctors’ prediction and AI-assisted doctors’ results (*P* < 0.05) (Fig. 3C).

## Discussion

The morbidity and mortality of cervical cancer remain high among women, especially in developing countries, despite the widespread availability of cervical cancer vaccines. [1, 2] And the age of diagnosis of cervical cancer tends to be younger. [3] Cervical cancer has always used clinical staging, until the FIGO staging in 2018 was slightly changed, and it was closer to TNM staging. [4, 5] The formulation and selection of cervical cancer treatment plan is significantly related to the stage. [4, 20, 22] However, the preoperative clinical staging and postoperative TNM staging are often inconsistent, because the final histology, grade and LNM can only be determined according the postoperative pathological results. Many cervical cancer patients with early clinical stage often showed postoperative para-aortic LNM. [6, 7] LNM is frequently associated with poor prognosis. [8] This study built an AI model on the basis of the postoperative TNM stage, which can precisely predict preoperative clinical stage, histology, grade and LNM at the same time.

Different from the endometrial cancer model previously published by our team [16], this study used retrospective data as the training set and prospective data as the independent validation set. In addition, the previous AI models for endometrial cancer predicted different targets by different models [16], while this paper used the identical AI model to predict multiple targets of cervical cancer simultaneously.

The accuracy of this cervical cancer AI model constructed with ANN algorithm in predicting stage, histology, grade and LNM were 75%, 95%, 86% and 76%, respectively. Compared with doctors’ prediction with or without AI assistance, the performance (accuracy and weighted F1 score) of this AI model is significantly better in predicting stage, histology, grade. When it comes to LNM, the accuracy of doctor’s predictions is a bit higher. This may be related to the small sample size of positive cases of LNM. In addition, the time consumption of this AI model is significantly less than that of doctor with or without AI assistance. Overall, the performance of this cervical cancer AI model is acceptable and applicable.

Multi-Task Learning (MTL) method as an improvement of traditional machine learning, which inspiration comes from the commonalities between multiple related tasks by related models and usually achieve better performance than Single-Task Learning (STL) [23–25]. There are already few proposed MTL based methods in medical prediction such as MTL based medical image diagnosis [26]. Our proposed method by sharing the parameters to build multiple classification ANN model, which can obtain more related information than STL models. For example, the stage, histology and differentiation grade of cervical cancer. The experiment illustrated that these tasks show better performance in MTL.

The findings of this study align with the broader literature on the application of AI in medical diagnostics. For example, research on emotional intelligence and self-efficacy has demonstrated the importance of integrating advanced predictive models in enhancing performance and decision-making [27]. Similarly, studies on emotional intelligence and learning strategies have highlighted the significant improvements in outcomes when multi-faceted approaches are employed [28]. Our AI model’s superior performance in multi-task learning further validates these findings by illustrating the efficacy of integrating complex, related tasks into a single predictive framework.

Furthermore, considering the recent evidence about the pattern of recurrence in cervical cancer patients after surgical treatment, and the comparison between minimally invasive approaches and open approach, [29] this MTL based AI model can simultaneously predict clinical stage, histology, grade and LNM for cervical cancer before surgery, which can help to develop the most beneficial treatment plan for patients. By predicting the histological types of cervical squamous cell carcinoma and adenocarcinoma before surgery, this MTL based AI model can help evaluate different surgical approaches for treatment of cervical cancer, considering various outcomes, possible complications, and each patient’s tailored treatment could be improved [30].

One of the strengths of this study is the application of the Multi-Task Learning (MTL) method, which improves traditional machine learning by leveraging the commonalities between multiple related tasks, often achieving better performance than Single-Task Learning (STL) [16–18]. Our method, which shares parameters to build multiple classification ANN models, can obtain more related information than STL models. This is evident in the improved performance of tasks such as stage, histology, and differentiation grade predictions for cervical cancer, which showed better results with MTL.

However, There are still some limitations. First, though the multi-task classification algorithm can share the information between different tasks, too many classes for prediction will lead the leak of data for specific class which will decrease the model performance. Second, prospective data validated model performance. However, multi-center, large sample size research will help improve the performance of this AI model. Moreover, this article used text information for prediction which lost a lot of image information. In the feature work, we will try to directly extract more information from pathological slides and imaging. Future studies should focus on refining the model for broader clinical applications, increasing the diversity of training datasets, and enhancing its adaptability to various clinical settings. Additionally, continuous feedback from clinical practice should be incorporated to ensure the model’s accuracy and reliability, ultimately improving personalized patient care and treatment outcomes. Further research should also explore the integration of image data to enhance prediction accuracy and model robustness.

## Conclusion

This study demonstrated that an MTL ANN model can simultaneously predict stage, histology, grade and LNM of cervical cancer preoperatively with minimal time consumption. This will help gynecologists to obtain more and better preoperative diagnostic information, so as to formulate more reasonable and personalized treatment plans for cervical cancer patients. But multicenter, multimodal and larger sample research and exploration are still needed. Future studies should focus on refining the model for broader clinical applications, increasing the diversity of training datasets, and enhancing its adaptability to various clinical settings. Continuous feedback from clinical practice should be incorporated to ensure the model’s accuracy and reliability, ultimately improving personalized patient care and treatment outcomes.

## Electronic supplementary material

Below is the link to the electronic supplementary material.

Supplementary Material 1

## Data Availability

The datasets used and/or analyzed during the current study are available from the corresponding author on reasonable request.
