# Correction: A multi-task learning based applicable AI model simultaneously predicts stage, histology, grade and LNM for cervical cancer before surgery

**DOI:** 10.1186/s12905-024-03435-y

**Published:** 2024-11-11

**Authors:** Zhixiang Wang, Huiqiao Gao, Xinghao Wang, Marcin Grzegorzek, Jinfeng Li, Hengzi Sun, Yidi Ma, Xuefang Zhang, Zhen Zhang, Andre Dekker, Alberto Traverso, Zhenyu Zhang, Linxue Qian, Meizhu Xiao, Ying Feng

**Affiliations:** 1 Department of Ultrasound, Beijing Friendship Hospital, Capital Medical University, Beijing, China; 2 Department of Obstetrics and Gynecology, Beijing Chao-Yang Hospital, Capital Medical University, Beijing, China; 3 Department of Radiation Oncology (Maastro), GROW-School for Oncology, Maastricht University Medical Centre+, Maastricht, The Netherlands; 4 Institute for Medical Informatics, University of Luebeck, 23562 Luebeck, Germany; 5 German Research Center for Artificial Intelligence, (DFKI), Lübeck, Germany

**Correction: BMC Women’s Health (2024) 24:425**

10.1186/s12905-024-03270-1

The original publication of this article [1] contained an incorrect affiliation for Zhixiang Wang. The incorrect and correct information is listed in this correction article, the original article has been updated.

Incorrect

—Zhixiang Wang^3^

^3^Department of Radiation Oncology (Maastro), GROW-School for Oncology, Maastricht University Medical Centre+, Maastricht, The Netherlands

Correct

—Zhixiang Wang^1,3^

^1^Department of Ultrasound, Beijing Friendship Hospital, Capital Medical University, Beijing, China

^3^Department of Radiation Oncology (Maastro), GROW- School for Oncology, Maastricht University Medical Centre+, Maastricht, The Netherlands

